# Supplementary material for: Direct bandgap emission from strain-doped germanium
Source: Nat Commun. 2024 Jan 19;15:618. doi: 10.1038/s41467-024-44916-w (PMC10799082; doi:10.1038/s41467-024-44916-w)
Supplement: Supplementary file 1 — Supplementary Information [file 41467_2024_44916_MOESM1_ESM.pdf]

Supplementary Information

Direct bandgap emission from strain-doped germanium

Lin-Ding Yuan[1†], Shu-Shen Li[1,2], and Jun-Wei Luo[1,2*]

[1]*State Key Laboratory for Superlattices and Microstructures, Institute of Semiconductors, Chinese Academy of Sciences, PO Box 912, Beijing 100083, China*

[2]*College of Materials Science and Opto-Electronic Technology, University of Chinese Academy of Sciences, Beijing, China*

†*Present address: Department of Materials Science and Engineering, Northwestern University, Evanston, Illinois 60208, USA*

[*] jwluo@semi.ac.cn

Supplementary Notes

1. Mixing of Γ and L valley in strain doped Ge

Supplementary Figure 1 shows the calculated Bloch characteristic of Ge:Li with 3.1 at.% of Li in Ge. One can observe that the CBM is an admixture of Γ (58.3%) and L (41.2%) even though the VBM is still dominated by bulk Γ (99.8%). This strong valley-mixing in the CBM renders the Ge:Li system to have a strong optical emission before the indirect-to-direct transition. The mixing of Γ and L for the CBM state can be understood from a perturbative view. Let H_0 be the Hamiltonian of the pure Ge, H_1 be the perturbative term introduced by inserted foreign atoms. This perturbative term H_1 violates the primitive translation symmetry of the pure Ge, which couples the original conduction band edge states $|\Gamma_c\rangle$ and $|L_c\rangle$ of the pure Ge. The CBM state of the Ge:Li system can then be approximated as a superposition state of $|\Gamma_c\rangle$ and $|L_c\rangle$ of the pure Ge:

$$|\mathrm{CBM}\rangle = |L_c\rangle + \frac{\langle \Gamma_\mathrm{c}|H_1|L_c\rangle}{E(L_c) - E(\Gamma_c)}|\Gamma_c\rangle$$

Because the energy separation between the $|\Gamma_c\rangle$ and $|L_c\rangle$ is small, the mixing is rather significant. This contrast to the situation for VBM where the $|\Gamma_v\rangle$ and $|L_v\rangle$ are far apart:

$$|\mathrm{VBM}\rangle = |\Gamma_\mathrm{v}\rangle + \frac{\langle L_\mathrm{v}|H_1|\Gamma_v\rangle}{E(\Gamma_v) - E(L_v)}|L_v\rangle \approx |\Gamma_\mathrm{v}\rangle$$

Consequently, the optical matrix element between CBM and VBM becomes finite, given that $\langle \Gamma_v|\hat{p}|\Gamma_\mathrm{c}\rangle = \Gamma_8^+ \otimes \Gamma_4^- \otimes \Gamma_7^- \sim \Gamma_1$.

2. Implanted atom prefers to stay at the tetrahedral interstitial sites (Td sites) in Si or Ge

In the main text, we have asserted that the implanted atoms stay at the tetrahedral interstitial sites (Td sites) based on some earlier studies. We now provide some additional justification for this assumption. Supplementary Table I gives the calculated formation energy of different defects for which implanted atoms would form. We find that the formation energy of Li at the Td site in Ge is 0.21 eV which is the smallest among others. This means Li favors staying at the Td site, which justifies our assumption. A similar conclusion could be reached for Ge:He, Ge:Ne, and Ge:Ar systems. But we do notice the interstitial C site that has similar or sometimes even lower energy competing with the Td site. For Ge:Ar, the system also likes to form Frenkel (F) defects. The form of these defects could be detrimental to achieving direct bandgap Ge.

3. Role of interstitial atoms distribution

In the main text, we have used the SQS structure for the evaluation of the ensuring changes of the direct and indirect bandgap of Ge:X (X=Li, He, Ne, Ar, Kr). To understand the impact of X distribution on the direct and indirect bandgap behavior. We now compare the direct and indirect bandgap, and the dipole matrix element of the SQS structure with other five randomly selected configures of $Ge_{32}Li_2$ systems. Supplementary Figure 2 shows our DFT calculated results for these six configurations. Clearly, all configures of the same Li concentration have very similar direct and indirect bandgap – difference is within 20 meV. Moreover, the optical dipole matrix elements of the direct bandgap transition are all optically allowed and differs only by 10-15%. These suggest the role of interstitial atoms distribution is subordinate at a few percent doping concentration.

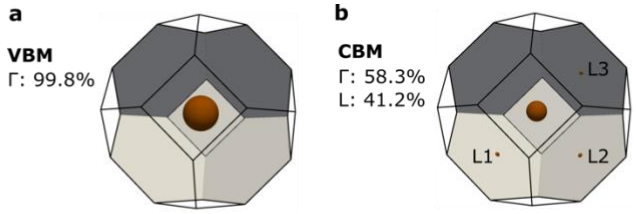

Supplementary Figure 1 | Bloch characteristic of Ge:Li with 3.1 at.% of Li. The block character of **(a)** the CBM and **(b)** the VBM of the Ge:Li system decomposed onto the pure Ge primitive cell.

$Ge_{32}Li_2$ (configurations)	Crystal structure	E_g^{Γ} (eV)	E_g^{L} (eV)	$E_P = 2P^2/m$ (eV)
SQS	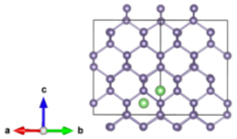	0.407	0.515	18.569
Random configuration 1	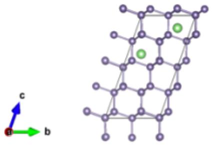	0.404	0.502	16.006
Random configuration 2	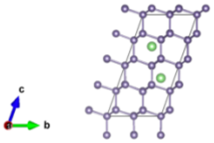	0.408	0.497	16.409
Random configuration 3	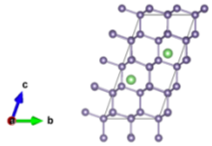	0.404	0.502	16.006
Random configuration 4	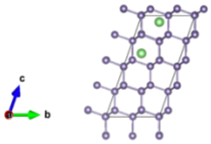	0.423	0.505	18.858
Random configuration 5	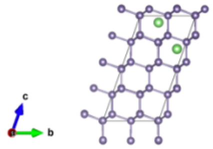	0.425	0.490	19.004

Supplementary Figure 2 | Role of Li distribution. DFT calculated direct bandgap, indirect bandgap and the corresponding fundamental bandgap dipole matrix element are examined for the SQS configure (used to obtain result of Fig. 1) and other five random configurations of in $Ge_{32}Li_2$ (6.25% at. Li in Ge).

Supplementary Table 1. Formation energy (eV) of defects in Si or Ge. Defect types of dopant atoms in Si or Ge include, Tetrahedral (Td), Hexagonal (Hex), Anti-bonding (A), Bond-center (B), Center of the second nearest neighbor (C), and Midway (M) sites, Substitutional (S) defect and a Frenkel (F) defect.

**System**	**Interstitial**						**Substitutional**	
	**Td**	**Hex**	**A**	**B**	**C**	**M**	**S**	**F**
**Si:Li**	0.508	0.952	0.508	2.688	0.508	0.952	7.931	2.508
**Ge:Li**	0.207	0.487	0.218	2.398	0.207	0.487	5.993	1.749
**Ge:He**	0.797	1.259	0.797	3.054	0.797	1.259	8.076	3.009
**Ge:Ne**	1.687	2.298	1.687	2.947	1.687	2.298	7.409	2.947
**Ge:Ar**	4.057	4.546	3.133	3.135	3.969	2.955	7.571	3.132
